# Desferrioxamine alleviates UHMWPE particle-induced osteoclastic osteolysis by inhibiting caspase-1-dependent pyroptosis in osteocytes

**DOI:** 10.1186/s13036-022-00314-8

**Published:** 2022-12-08

**Authors:** Shenli Zhao, Chen Ge, Yao Li, Leilei Chang, Zhou Dan, Yihui Tu, Lianfu Deng, Hui Kang, Changwei Li

**Affiliations:** 1 Department of Orthopedics, Yangpu Hospital, Tongji University School of Medicine, Shanghai, China; 2 Department of Orthopedics, Shanghai Key Laboratory for the Prevention and Treatment of Bone and Joint Diseases, Shanghai Institute of Traumatology and Orthopedics, Ruijin Hospital, Shanghai Jiao Tong University School of Medicine, No.197, Ruijin 2Nd Road, Shanghai, 200025 China; 3 Department of Orthopedic Surgery, Ruijin Hospital, Shanghai Jiao Tong University School of Medicine, Shanghai, China; 4 Nanjing Medical University School of Medicine, Nanjing, China; 5 Department of Orthopedics, Shanghai Tenth People’s Hospital, Tongji University School of Medicin, No. 301 Middle Yanchang Road, Shanghai, 200072 China

**Keywords:** Wear particles, Osteolysis, Osteocytes, Pyroptosis, Osteoclasts

## Abstract

**Background:**

Cell death and inflammation are the two important triggers of wear particle-induced osteolysis. Particles, including cobalt-chromium-molybdenum and tricalcium phosphate, have been reported to induce pyroptosis in macrophages and osteocytes. Although macrophage pyroptosis facilitates osteoclastic bone resorption and osteolysis, whether osteocyte pyroptosis is involved in osteoclastic osteolysis still needs further investigation. Desferrioxamine (DFO), an FDA-approved medication and a powerful iron chelator, has been proven to reduce ultrahigh-molecular-weight polyethylene (UHMWPE) particle-induced osteolysis. However, whether DFO can ameliorate UHMWPE particle-induced osteolysis by decreasing pyroptosis in osteocytes is unknown.

**Results:**

A mouse calvarial osteolysis model and the mouse osteocyte cell line MLO-Y4 was used, and we found that pyroptosis in osteocytes was significantly induced by UHMWPE particles. Furthermore, our findings uncovered a role of caspase-1-dependent pyroptosis in osteocytes in facilitating osteoclastic osteolysis induced by UHMWPE particles. In addition, we found that DFO could alleviate UHMWPE particle-induced pyroptosis in osteocytes in vivo and in vitro.

**Conclusions:**

We uncovered a role of caspase-1-dependent pyroptosis in osteocytes in facilitating osteoclastic osteolysis induced by UHMWPE particles. Furthermore, we found that DFO alleviated UHMWPE particle-induced osteoclastic osteolysis partly by inhibiting pyroptosis in osteocytes.

**Graphical Abstract:**

Schematic of DFO reducing UHMWPE particle-induced osteolysis by inhibiting osteocytic pyroptosis. Wear particles, such as polymers, generated from prosthetic implant materials activate canonical inflammasomes and promote the cleavage and activation of caspase-1. This is followed by caspase-1-dependent IL-β maturation and GSDMD cleavage. The N-terminal fragment of GSDMD binds to phospholipids on the cell membrane and forms holes in the membrane, resulting in the release of mature IL-β and inflammatory intracellular contents. This further facilitates osteoclastic differentiation of BMMs, resulting in excessive bone resorption and ultimately leading to prosthetic osteolysis. DFO reduces UHMWPE particle-induced osteolysis by inhibiting osteocytic pyroptosis.

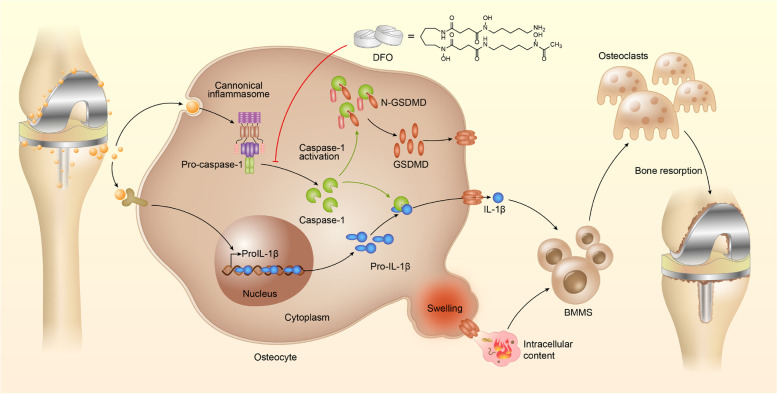

**Supplementary Information:**

The online version contains supplementary material available at 10.1186/s13036-022-00314-8.

## Background

Late aseptic loosening, which is one of the major complications of joint arthroplasty, is mostly produced by wear particles generated from prosthetic implant materials such as polymers, metal and bone cements [1]. This wear debris induces a series of tissue inflammatory responses in a wide variety of peri-implant cells, which in turn directly or indirectly stimulate osteoclast attachment, differentiation, activation and maturation, leading to prosthetic osteolysis and implant failure [2–4].

Normal bone remodeling maintains constant bone mass by an orchestrated balance between the destruction of old bone by osteoclasts and the rebuilding of bone by osteoblasts [5]. Osteoclasts are multinucleated giant cells originating from bone myelomonocytes (BMMs) through a differentiation process that is mainly governed by two key cytokines: macrophage colony-stimulating factor (M-CSF) and receptor activator of nuclear factor kappa B ligand (RANKL) [6]. In localized inflammatory bone resorption, such as periprosthetic osteolysis, foreign wear debris activates macrophages, phagocytes and T lymphocytes, resulting in the production of high concentrations of chemokines and cytokines, such as M-CSF, IL-1, IL-6, PGE2 and TNF-α, which lead to an increase in RANKL and/or have direct effects on osteoclastogenesis and the resorption of adjacent bony structures [7–9].

Osteocytes, which make up over 90% of all bone cells, are derived from osteoblasts on the bone surface. These cells are embedded in lacunae within the mineralized matrix and form a syncytial network to communicate with neighboring osteocytes and other cells at the bone surface via cell processes within canaliculi [1, 10]. Osteocytes are considered to be master of controlling bone remodeling and mineral homeostasis by osteoblasts and osteoclasts [11–13]. Targeted ablation of osteocytes was shown to induce osteoporosis with the deterioration of bone microstructure and mechanotransduction [14]. Several reports showed that osteocyte death by apoptosis was implicated in pathological conditions that correlated with bone loss [15, 16]. Although the interaction between osteocytes and implant debris has not been well characterized, recent studies have suggested that osteocytes may be involved in aseptic loosening by affecting osteoclast generation, which causes bone resorption. Osteocyte-like cells exposed to polyethylene and metal wear particle types showed upregulated expression of the osteoclastic markers RANKL and M-CSF [17]. In addition to the direct resorptive effects, polyethylene wear particles upregulate the expression of proinflammatory cytokines, including IL-8 [18], IL-6 and TNF-α [17], in human primary osteocyte-like cells, which subsequently form an extracellular inflammatory microenvironment and have been shown to exert osteoclastogenic effects and drive osteolysis [19–21]. In addition, exogenous tricalcium phosphate (TCP) wear particle implantation could trigger death in osteocytes and the production of osteoclastic mediators, including RANKL, M-CSF, TNF-α, IL-6 and IL-1β [1]. This finding suggests that osteocytes likely undergo a death pathway, which may be involved in the onset of osteoclastic osteolysis induced by wear particles.

Pyroptosis is a form of proinflammatory programmed cell death [22] that has the biochemical and morphological characteristics of necrosis and apoptosis, but unlike apoptosis or necrosis [23], pyroptosis results in the release of cytokines that activate proinflammatory immune cell mediators [24–26]. In response to signals from pathogens or many exogenous stimuli, caspase-1 is activated during pyroptosis by a large supramolecular complex known as the pyroptosome and subsequently processes the proforms of the inflammatory cytokines IL-1β and IL-18 into their active forms to trigger or aggravate inflammatory responses [27–29]. Therefore, pyroptosis not only leads to cell death but also may play an important role in the cascade of reactions that lead to tissue damage. Regarding the role of osteocytes in wear debris-induced osteolysis, it has been reported that exposure to CoCrMo particles induces pyroptosis in macrophages, which promotes the release of cytokines such as IL-18, IL-1β and HMGB1 and the subsequent formation of an extracellular inflammatory microenvironment that leads to osteoclastic bone resorption [30]. In addition, pyroptotic death in osteocytes has been observed in TCP particle-induced mouse calvarial osteolysis [31]. However, whether osteocytic pyroptosis is involved in osteoclastic osteolysis still needs further elucidation.

By understanding the pathogenesis of periprosthetic osteolysis, some effective preventative and nonsurgical interventions focusing on inhibiting inflammation and targeting osteoclasts have been introduced [32]. Recently, TNF-α and IL-1 antagonists have demonstrated variable efficacy in alleviating aseptic loosening, whereas inhibiting TNF-α and IL-1 may lead to blunted reactions to harmful agents, including bacteria and other infective agents [33]. Early postoperative systemic administration of bisphosphonates can decrease the risk of aseptic loosening by inhibiting osteoclast-mediated bone resorption [34]. However, bisphosphonates are unsuccessful in treating inflammatory conditions [33]. Furthermore, it has been reported that long-term administration of bisphosphonates may be associated with bone necrosis and atypical fractures in long bones [35]. Thus, identifying more suitable strategies for wear particle-induced osteolysis is urgently needed.

DFO, an FDA-approved medication and a powerful iron chelator, is widely used as a therapeutic agent for treating iron overload-related diseases [36]. Recently, considerable efforts have been directed toward using DFO in orthopedic pharmacology, not only for its role in bolstering bone repair and regeneration in various bone defect models by stimulating angiogenesis and osteoblast differentiation [37] but also for its function in inhibiting osteoclast formation [38]. In addition, our previous study revealed that DFO could reduce UHMWPE particle-induced osteolysis by inhibiting inflammatory osteoclastogenesis [39]. However, the intricate mechanism by which DFO impairs osteoclastic osteolysis induced by UHMWPE particles remains largely unknown.

In the present study, we identified that caspase-1-dependent pyroptosis in osteocytes facilitated osteoclastic osteolysis induced by UHMWPE particles. Furthermore, we found that DFO alleviated UHMWPE particle-induced osteoclastic osteolysis partly by inhibiting pyroptosis in osteocytes.

## Results

### Pyroptosis is accompanied by calvarial osteolysis induced by UHMWPE particles

**Fig. 1:**
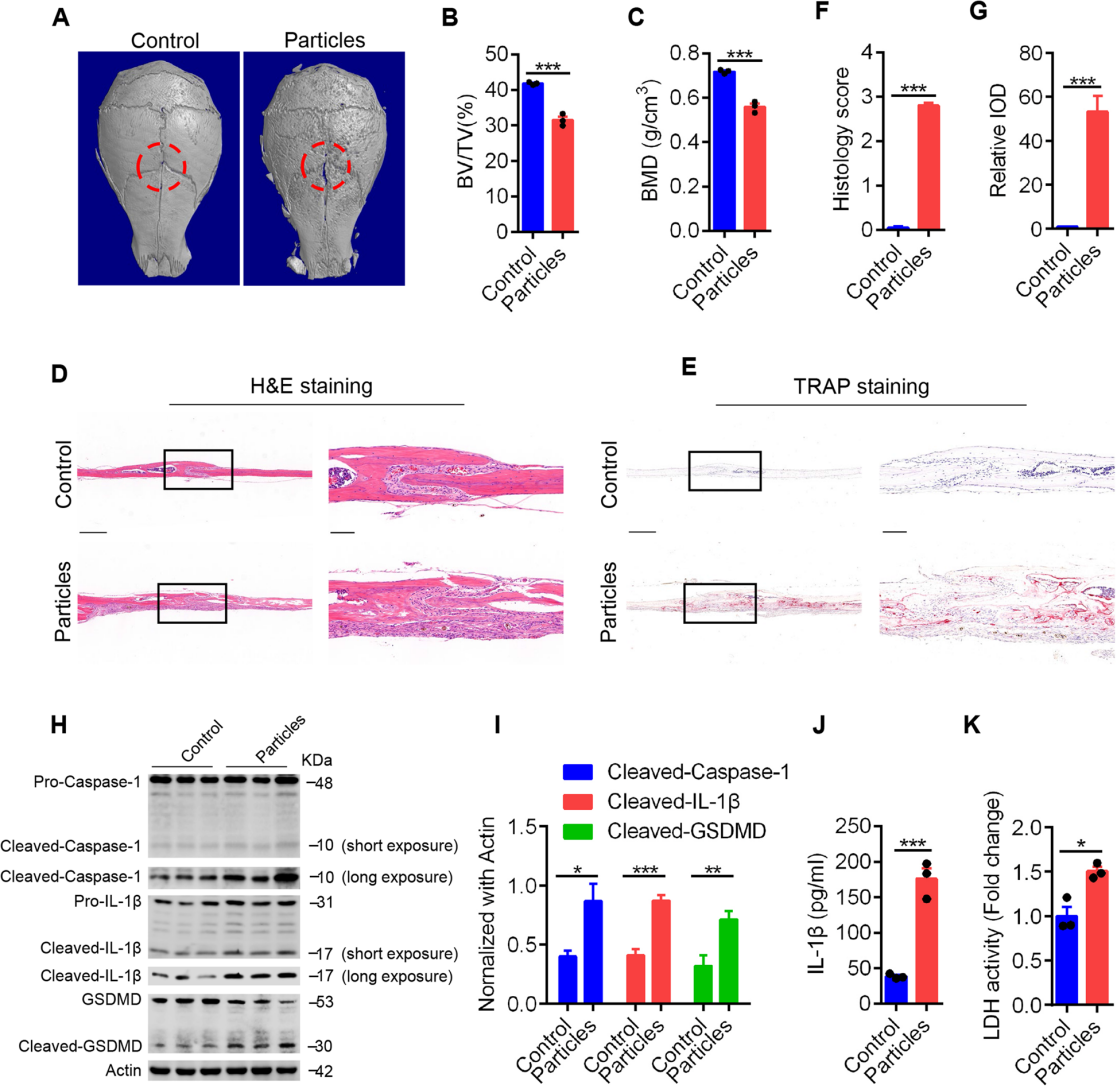
Pyroptosis is accompanied by calvarial osteolysis induced by UHMWPE particles. **A** Representative micro-CT three-dimensional reconstructed images of calvarial (*n* = 3). Dotted red lines indicate the region of interest. (B&C) BMD and BV/TV in the region of interest shown in (**A**) were measured. (D&E) H&E staining (**D**) and TRAP staining (**E**) of longitudinal sections of calvarial (*n* = 3), scales bars represent 400 μm. The rightmost pictures designate the larger magnification of the regions shown in inset, scales bars represent 100 μm. **F **& **G** Bar charts showing the histological score and integrated optical density (IOD) value of TRAP positive area shown in (**D**&**E**). (H) The protein level of caspase-1, IL-1β, and GSDMD detected by western blot analysis. **I** Bar charts showing the densitometric analysis of western blot shown in (**H**). **J** The protein level of IL-1β detected by ELISA in culture supernatant of calvarial tissues. **K** The relative LDH activity in culture supernatant of calvarial tissues. *P < *0.05*, **P < *0.01*, ***P < *0.001*. *P*-values were analyzed by two-tailed *t* tests. All data are representative of two to three independent experiments

To investigate whether pyroptosis in osteocytes is involved in UHMWPE particle-induced osteolysis, a murine calvarial osteolysis model induced by UHMWPE particles was used as previously described [39]. Microcomputed tomography (CT) scanning showed extensive resorption pits present in the calvarial in the UHMWPE particle group (Fig. 1A). Further trabecular architectural parameter analysis showed decreased bone mineral density (BMD) and bone volume/tissue volume (BV/TV) in calvarial after UHMWPE particle treatment (Fig. 1B & C). Hematoxylin and eosin (H&E) staining revealed that UHMWPE particle stimulation significantly increased inflammatory cell infiltration and osteolysis (Fig. 1D & F). Tartrate-resistant acid phosphatase (TRAP) staining showed an increased number of osteoclasts lining the eroded bone surface (Fig. 1E & G). Along with inflammatory osteolysis, western blot analysis showed that there were increased protein levels of cleaved caspase-1 and IL-1β with molecular weights of 10 kDa and 17 kDa, respectively (Fig. 1H & I). In addition, the results revealed that the pyroptosis effector gasdermin D (GSDMD) was cleaved concomitantly with the release of mature IL-1β and increased lactic dehydrogenase (LDH) activity (Fig. 1I-K). Therefore, these data demonstrated that pyroptosis was activated in UHMWPE particle-induced calvarial osteolysis.

### Pyroptosis facilitates UHMWPE particle-induced calvarial osteolysis

**Fig. 2:**
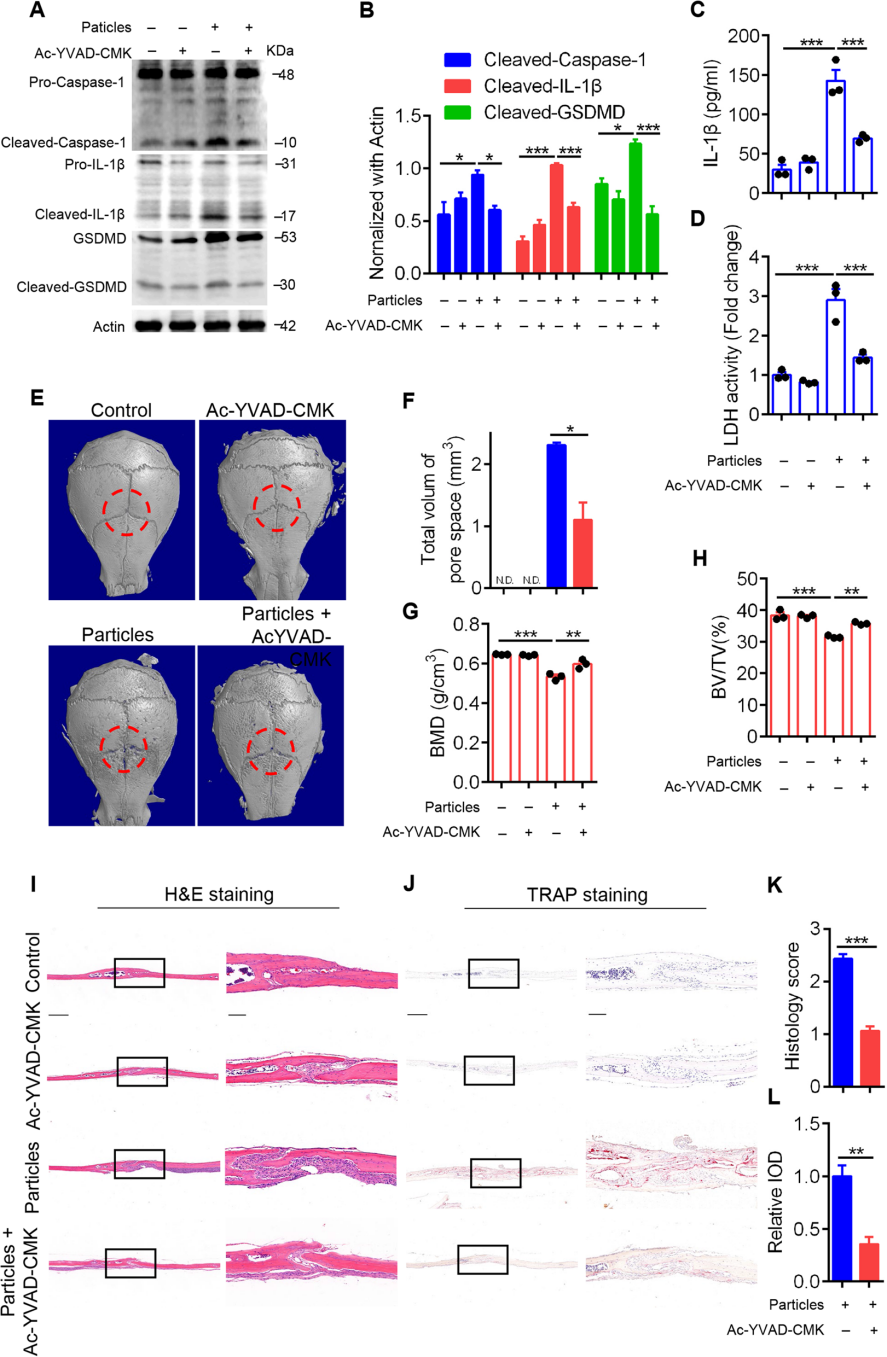
Pyroptosis facilitates UHMWPE particle-induced calvarial osteolysis. **A** western blot analysis of caspase-1, IL-1β, and GSDMD in calvarial treated with UHMWPE particles and/or Ac-YVAD-CMK. **B** Bar charts showing the densitometric analysis of western blot shown in **A**. **C**&**D** The protein level of IL-1β and the relative LDH activity in culture supernatant of calvarial tissues (*n* = 3). **E** Representative micro-CT three-dimensional reconstructed images of calvarial. Dotted red lines indicate the region of interest (*n* = 3). **F** Bar charts showing the total volume of pore space in calvarial shown in **E**. **G**&**H** BMD and BV/TV in the region of interest shown in (**E**) were measured (*n* = 3). **I**&**J** H&E staining (**I**) and TRAP staining (**J**) of longitudinal sections of calvarial (*n* = 3), scales bars represent 400 μm. The rightmost pictures designate the larger magnification of the regions shown in inset, scales bars represent 100 μm. **K**&**L** Bar charts showing the histological score and integrated optical density (IOD) value of TRAP positive area shown in (**I**&**J**). *P < *0.05*, **P < *0.01*, ***P < *0.001*. *P*-values were analyzed by one-way ANOVA in (**C**&**D**, **G**&**H**), two-tailed *t* tests in (**F**, **K**&**L**). All data are representative of two to three independent experiments

Having observed increased pyroptosis activation in UHMWPE particle-induced calvarial osteolysis, we next sought to determine whether pyroptosis plays a pathological role in UHMWPE particle-induced osteolysis. Since caspase-1 plays a critical role in inducing pyroptosis by cleaving GSDMD and IL-1β and leading to mature IL-1β and secretion, we inhibited pyroptosis activation by blocking caspase-1 activity with Ac-YVAD-CMK, a highly selective caspase-1 inhibitor [40], in the UHMWPE particle-induced murine calvarial osteolysis model. The western blot results showed that UHMWPE particles robustly induced caspase-1 activation, as evidenced by increased caspase-1 cleavage, whereas this cleavage was markedly inhibited by Ac-YVAD-CMK (Fig. 2A & B). Consistently, we observed impaired IL-1β cleavage and mature IL-1β release, as well as LDH activity, in the culture supernatant of calvarial tissues (Fig. 2B-D). Isolated calvarials were analyzed by micro-CT scanning. The results showed that extensive bone resorption was present in the calvarial in the UHMWPE particle group, as evidenced by an increased volume of pore space (Fig. 2E & F). However, UHMWPE particle-induced bone resorption was significantly attenuated by Ac-YVAD-CMK (Fig. 2E & F). Moreover, the rescued BMD and BV/TV further confirmed the protective effect of inhibiting pyroptosis on UHMWPE particle-induced osteolysis (Fig. 2G & H).

Next, histomorphological analysis was performed to evaluate the effect of pyroptosis inhibition on UHMWPE particle-induced osteolysis. H&E staining showed that UHMWPE particles significantly induced inflammatory responses and prominent osteolysis, whereas these processes were markedly dampened by Ac-YVAD-CMK (Fig. 2I & K). Consistently, TRAP staining showed that Ac-YVAD-CMK significantly decreased the number of osteoclasts lining the eroded bone surface in response to UHMWPE particles (Fig. 2J & L). Collectively, these data demonstrated that pyroptosis facilitated UHMWPE particle-induced calvarial osteolysis.

### UHMWPE particles induce pyroptotic death in osteocytes

**Fig. 3:**
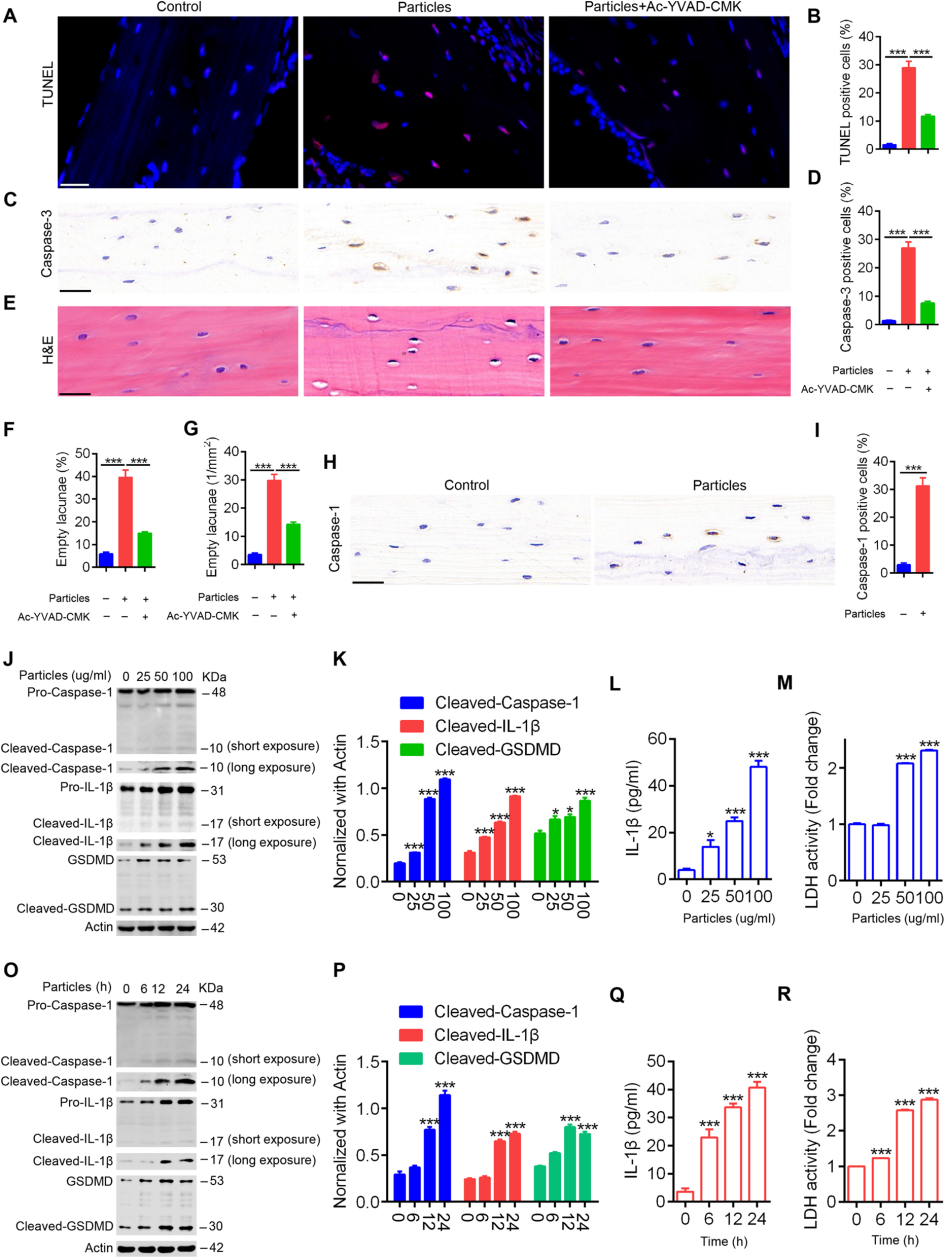
UHMWPE particles induce pyroptosis in osteocytes. **A**, **C**, **E** TUNEL staining (**A**), caspase-3 IHC staining (**C**) and H&E staining (**E**) of longitudinal sections of calvarial (*n* = 3), scales bars represent 25 μm. **B**&**D** Bar charts showing the percentage of TUNEL or caspase-3 positive cells shown in (**A**&**C**). (F&G) Bar charts showing the percentage of osteocytes with empty lacunae and the number of osteocytes with empty lacunae per mm^2^ shown in (**E**). **H** caspase-1 IHC staining of longitudinal sections of calvarial (*n* = 3), scales bars represent 25 μm. **I** Bar charts showing the percentage of caspase-1 positive cells shown in (**H**). **J**&**O** Western blot analysis of caspase-1, IL-1β, and GSDMD in MLO-Y4 cells induced by different doses of UHMWPE particles for 24 h (**J**) or by 50 μg/ml UHMWPE particles for different hours (**O**). **K**&**P** Bar charts showing the densitometric analysis of western blot shown in (**J**&**O**). **L**&**Q** The concentration of IL-1β in culture supernatant of MLO-Y4 cells induced by UHMWPE particles. **M**&**R** The relative LDH activity in culture supernatant of MLO-Y4 cells induced by UHMWPE particles. *P < *0.05*, ***P < *0.001*. *P*-values were analyzed by one-way ANOVA in (**B**, **D**, **F**, **G**, **K**, **L**, **M**, **P**, **Q**, **R**), and two-tailed *t* tests in (**I**). All data are representative of two to three independent experiments

Our results showed that UHMWPE implantation induced pyroptosis in calvarial tissues. Since osteocytes make up over 90% of all bone cells, we next examined pyroptosis in osteocytes in response to UHMWPE particles. The immunostaining results revealed that TUNEL- and caspase-3-positive osteocytes were significantly increased in calvarial after UHMWPE implantation (Fig. 3A-D). This result suggested that UHMWPE particles induced osteocyte death in vivo. Pyroptosis is uniquely dependent on the activation of caspase-1, and our results further showed an increased level of caspase-1 in calvarial osteocytes after UHMWPE particle implantation (Fig. 3H & I). Accompanied by pyroptotic death, an increase in empty osteocyte lacunae was detected in the mouse osteolysis model induced by TCP particles[31]. We found that UHMWPE particle implantation significantly increased empty osteocyte lacunae. The number of empty lacunae in the UHMWPE particle group increased approximately 6.7-fold compared with that in the sham group, and there were 29.75 ± 2.175 empty lacunae per mm^2^, whereas in the sham group, there were only 3.5 ± 0.6455 empty osteocyte lacunae per mm^2^. However, these effects were significantly inhibited by Ac-YVAD-CMK (Fig. 3E-G). Collectively, these data indicated that UHMWPE particles induced pyroptotic death in osteocytes in vivo.

We next examined pyroptosis in osteocytes in response to UHMWPE particles in vitro. As shown in Fig. 3J, K, O and P, UHMWPE particles induced the expression of caspase-1 and IL-1β, as well as GSDMD cleavage, in a dose- and time-dependent manner. Additionally, a gradual increase in the levels of IL-1β and the activity of LDH in the culture medium of MLO-Y4 osteocytes was also observed in response to UHMWPE particle stimulation (Fig. 3L & M, Q & R). These data demonstrated that UHMWPE particles induced pyroptosis in osteocytes in vitro.

### Osteocytic pyroptosis facilitates osteoclastic bone resorption

**Fig. 4:**
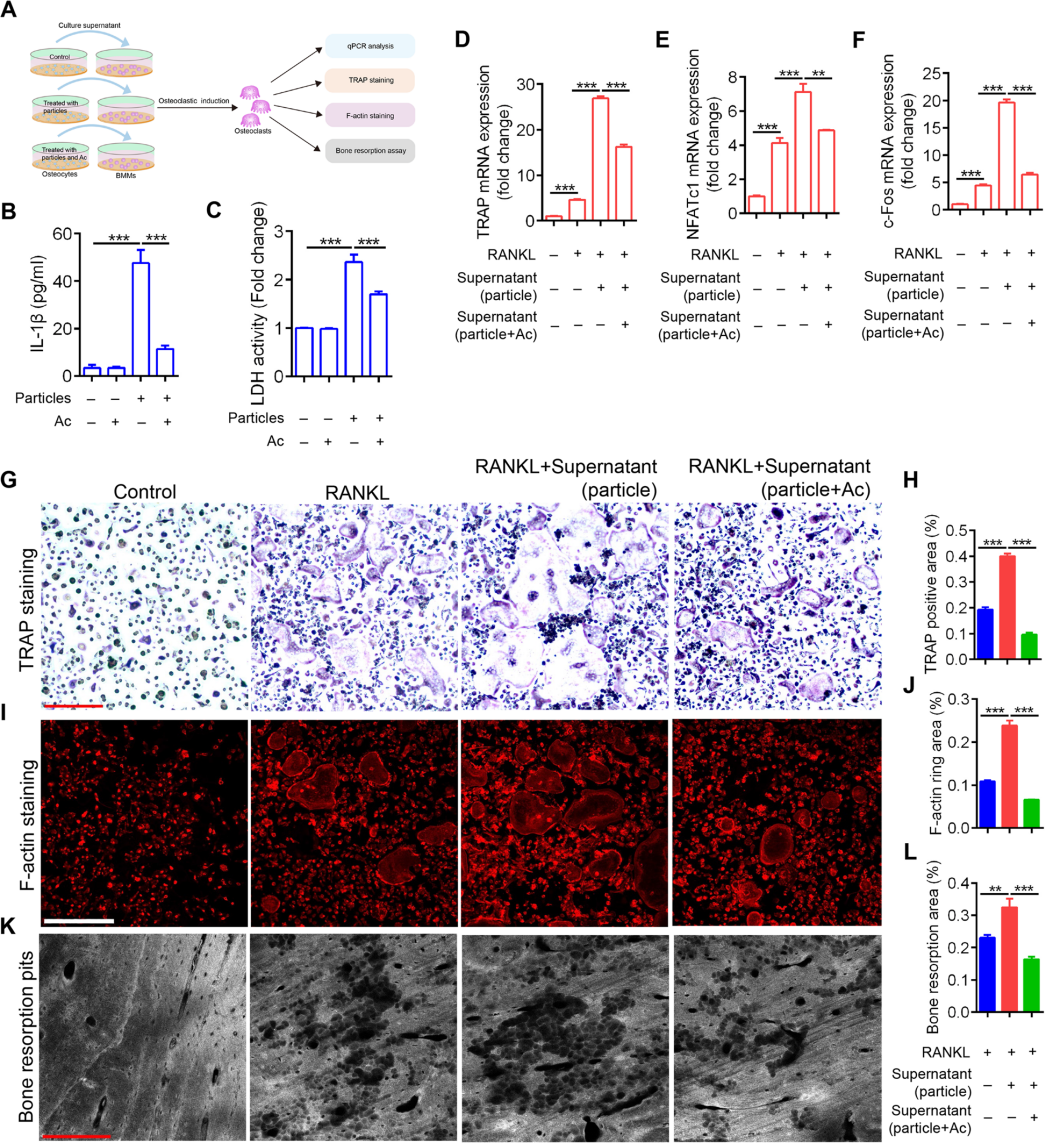
Osteocytic pyroptosis facilitates osteoclastic bone resorption. **A** Schematic of osteoclastic differentiation of BMMs induced by culture supernatant of osteocytes stimulated with UHMWPE particles and/or Ac-YVAD-CMK. Ac represents Ac-YVAD-CMK. **B** The concentration of IL-1β in culture supernatant of MLO-Y4 cells induced by 50 μg/ml UHMWPE particles and/or Ac-YVAD-CMK. **C** The relative LDH activity in culture supernatant of MLO-Y4 cells induced by 50 μg/ml UHMWPE particles and/or Ac-YVAD-CMK. **D**-**F** The relative mRNA expression of TRAP, NFATc1 and c-Fos in BMMs-derived osteoclasts treated with RANKL and/or culture supernatant of UHMWPE and/or Ac-YVAD-CMK-treated MLO-Y4 cells. **G**, **I**, **K** TRAP staining (**G**), F-actin ring formation (**I**) and bone resorption assay (**K**) of BMMs-derived osteoclasts treated with RANKL and/or culture supernatant of UHMWPE and/or Ac-YVAD-CMK-treated MLO-Y4 cells. **H**, **J**, **L** Bar charts showing the percentage of TRAP positive area, F-actin ring area and bone resorption area shown in (**G**, **I**, **K**). **P < *0.05*, ***P < *0.001*. *P*-values were analyzed by one-way ANOVA. All data are representative of two to three independent experiments

Pyroptotic death in osteocytes and osteoclastic bone resorption induced by UHMWPE particles were observed. We next examined the effect of osteocyte pyroptosis on osteoclastic bone resorption. Osteocytes were cultured with UHMWPE and/or Ac-YVAD-CMK for 24 h. Then, the culture medium was collected and incubated with BMMs, and osteoclast differentiation was assayed (Fig. 4A). First, we found that Ac-YVAD-CMK treatment significantly decreased the concentration of IL-1β and reduced the activity of LDH in the supernatant of MLO-Y4 cells (Fig. 4B & C). In addition, compared with that in the vehicle control, the supernatant of osteocytes treated with UHMWPE particles significantly increased osteoclastic differentiation, as evidenced by the expression of osteoclastic differentiation-related genes, including TRAP, nuclear factor of activated T cells c1 (Nfatc1) and c-Fos, as well as demonstrated by the TRAP staining assay (Fig. 4D-H). In addition, the F-actin ring and resorption pit formation assay further demonstrated the advanced effect of osteocytic pyroptosis on mature osteoclast formation and osteoclastic bone resorption (Fig. 4I & L). However, pretreatment of osteocytes with the caspase-1 inhibitor Ac-YVAD-CMK significantly impaired these processes (Fig. 4D-L). Taken together, these data indicated that pyroptosis in osteocytes facilitates osteoclastic bone resorption.

### DFO ameliorates UHMWPE particle-induced calvarial osteolysis by decreasing pyroptosis

**Fig. 5:**
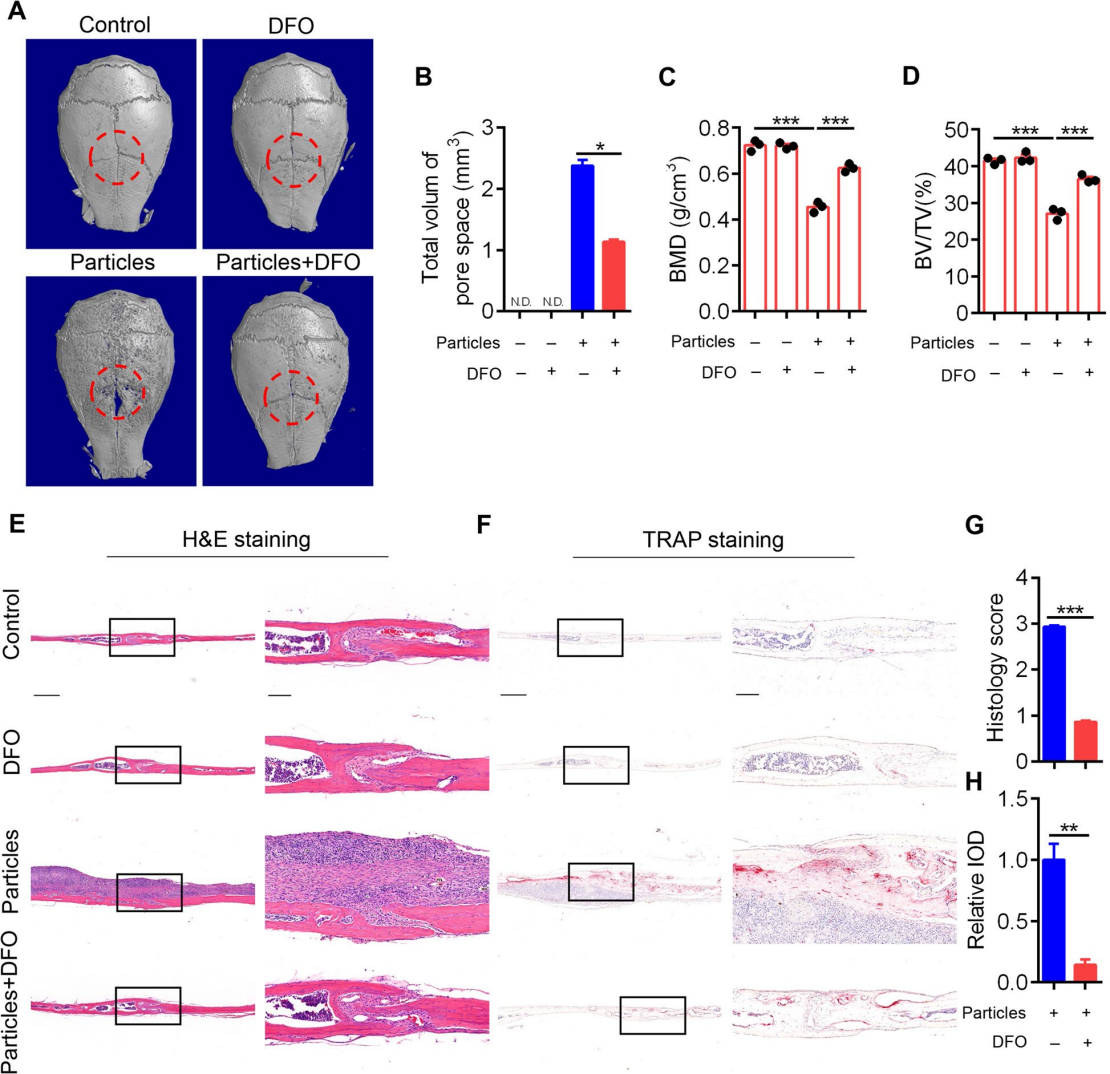
DFO alleviates UHMWPE particle-induced calvarial osteolysis. **A** Representative micro-CT three-dimensional reconstructed images of calvarial. Dotted red lines indicate the region of interest (*n* = 3). (**B**) Bar charts showing the total volume of pore space in calvarial shown in (**A**). **C**&**D** BMD and BV/TV in the region of interest of (**A**) were measured (*n* = 3). **E**&**F** H&E staining (**E**) and TRAP staining (**F**) of longitudinal sections of calvarial (*n* = 3). Scales bars represent 400 μm. The rightmost pictures designate the larger magnification of the regions shown in inset. Scales bars represent 100 μm. **G**&**H** Bar charts showing the histological score and integrated optical density (IOD) value of TRAP positive area shown in (**E**&**F**). *P < *0.05*, **P < *0.05*, ***P < *0.001*. *P*-values were analyzed by one-way ANOVA in (**C**&**D**), two-tailed *t* tests in (**B**, **G**&**H**). All data are representative of two to three independent experiments

Our previous study reported that DFO reduced UHMWPE particle-induced inflammatory responses and osteoclastic bone resorption [39]. Here, we examined whether DFO could alleviate UHMWPE particle-induced calvarial osteolysis by decreasing pyroptosis in osteocytes. Consistently, our present data further confirmed the protective role of DFO in UHMWPE particle-induced calvarial osteolysis. The micro-CT results revealed that DFO treatment significantly decreased the number of resorption pits and blocked the decrease in calvarial BMD and bone volume induced by UHMWPE particles (Fig. 5A-D). H&E staining and TRAP staining further demonstrated that DFO treatment significantly decreased inflammatory responses and osteoclastic bone resorption (Fig. 5E-H).

**Fig. 6:**
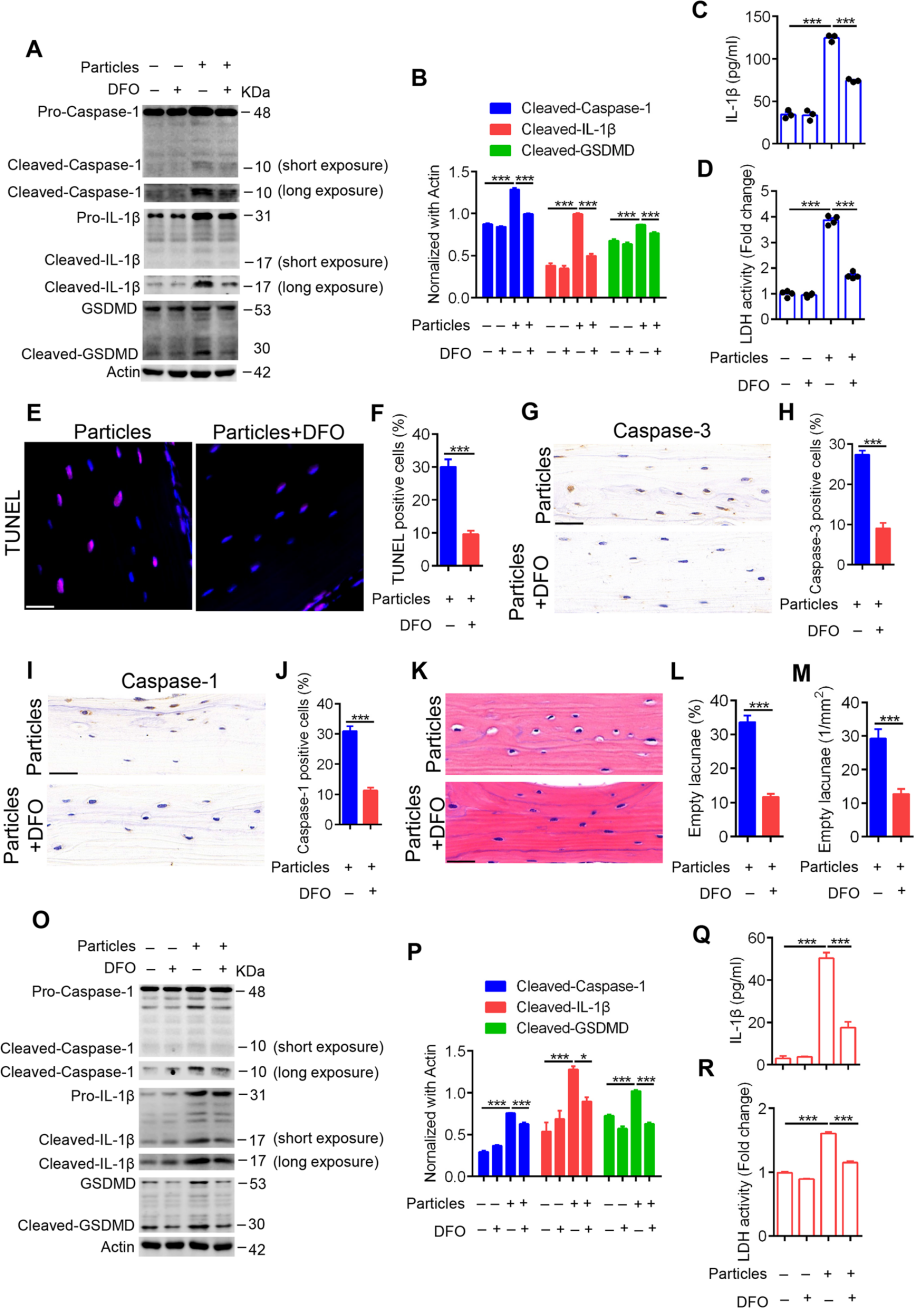
DFO alleviates UHMWPE particle-induced osteocytic pyroptosis. **A** Western blot analysis of caspase-1, IL-1β, and GSDMD in calvarial treated with UHMWPE particles and/or DFO. **B** Bar charts showing the densitometric analysis of western blot shown in (**A**). **C**&**D** The protein level of IL-1β and the relative LDH activity in culture supernatant of calvarial tissues (*n* = 3 or 4). **E**, **G**, **I**, **K** TUNEL staining, caspase-3 and caspase-1 IHC staining and H&E staining of longitudinal sections of calvarial (*n* = 3), scales bars represent 25 μm. **F**, **H**, **J** Bar charts showing the percentage of TUNEL, caspase-3 or caspase-1 positive cells shown in (**E**, **G**, **I**). **L**&**M** Bar charts showing the percentage of osteocytes with empty lacunae and the number of osteocytes with empty lacunae per mm^2^ shown in (**K**). **O** Western blot analysis of caspase-1, IL-1β, and GSDMD in MLO-Y4 cells induced by UHMWPE particles and/or DFO. **P** Bar charts showing the densitometric analysis of western blot shown in (**O**). **Q**&**R** The protein level of IL-1β and the relative LDH activity in culture supernatant of MLO-Y4 cells. *P < *0.05*, ***P < *0.001*. *P*-values were analyzed by one-way ANOVA in (**B**-**D**, **P**-**R**) and two-tailed *t* tests in (**F**, **H**, **J**, **L**, **M**). All data are representative of two to three independent experiments

Accompanied by the alleviation of osteolysis, UHMWPE particle-induced pyroptosis in osteocytes was significantly inhibited by DFO. The cleavage of caspase-1, IL-1β and GSDMD, as well as the concentrations of IL-1β and activity of LDH in murine calvarials induced by UHMWPE particles, were markedly decreased by DFO treatment (Fig. 6A-D). Further examination showed that the osteocyte death induced by UHMWPE particles was significantly inhibited by DFO, as evidenced by the percentage of TUNEL- and caspase-3-positive osteocytes in calvarials (Fig. 6E-H). Moreover, caspase-1 expression was significantly decreased by DFO treatment (Fig. 6I & J). In addition, H&E staining showed that empty osteocyte lacunae were significantly decreased by DFO treatment (Fig. 6K-M). Collectively, these data suggested a protective role of DFO against pyroptotic death of osteocytes induced by UHMWPE particles in vivo.

**Fig. 7:**
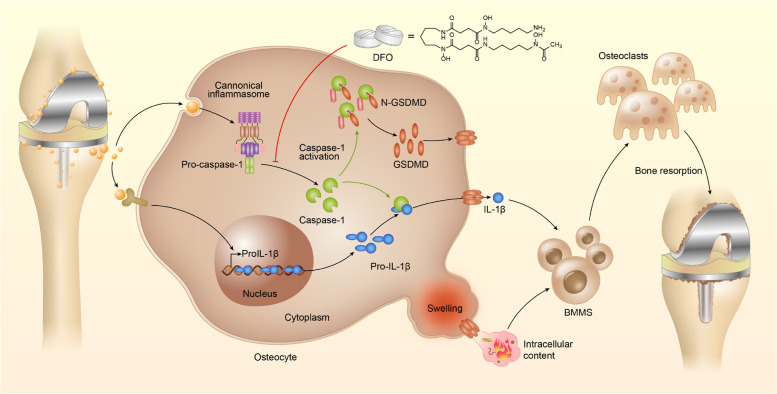
Schematic of DFO reduces UHMWPE particle-induced osteolysis by restraining osteocytic pyroptosis. The wear particles, generate from prosthetic implants materials, induce the activation of canonical inflammasomes and promote the cleavage and activation of caspase-1. This is followed by caspase-1-dependent IL-β maturation and GSDMD cleavage. The N-terminal fragment of GSDMD binds phospholipids on the cell membrane and further forms holes in the membrane, resulting in the release of mature IL-β and inflammatory intracellular content. Which further facilitate osteoclastic differentiation of BMMs, results in excessive bone resorption and ultimately leads to prosthetic osteolysis. DFO reduces UHMWPE particle-induced osteolysis by restraining osteocytic pyroptosis

Consistently, the protective role of DFO in UHMWPE particle-induced pyroptosis in osteocytes was further confirmed in vitro. As shown in Fig. 6O-R, pretreatment with 50 μM DFO significantly inhibited the cleavage of caspase-1, IL-1β and GSDMD at the cellular level and decreased the release of mature IL-1β and LDH activity in the culture supernatant. Taken together, these data demonstrated that DFO could ameliorate UHMWPE particle-induced osteoclastic bone resorption by decreasing the pyroptotic death of osteocytes (Fig. 7).

## Discussion

Joint replacement surgery is a successful treatment, as it restores mobility, diminishes pain and improves the overall quality of life for millions of people. Unfortunately, over time, wear of these prostheses generates debris, which is released into the joint space, embeds into the surrounding synovial tissues and subsequently induces a series of tissue immune responses that directly or indirectly stimulate osteoclast attachment, differentiation, activation and maturation, which ultimately lead to prosthetic osteolysis and implant failure [1–4].

Osteocytes, which are the most abundant cells in bone, are terminally differentiated cells of the osteoblast lineage that have become embedded in mineralized matrix and may send signals that regulate bone modeling and remodeling [12]. Although the interaction between osteocytes and implant debris has not been well characterized, recent studies have suggested that osteocytes may be involved in aseptic loosening by affecting osteoclast generation and/or recruitment that causes bone resorption [41]. In addition to inducing osteocytes to release proinflammatory mediators, such as PGE2, NO, IL-1β and TNF-α, wear particle stimulation leads to considerable death in osteocytes, which may have a causal relationship with osteoclast activation and local bone resorption [1]. Therefore, fully understanding the interaction between osteocytes and implant particles and developing innovative therapies targeting osteocyte death and inflammatory responses appear to be ideal alternatives for the treatment of wear particle-induced aseptic loosening.

Pyroptosis is a form of proinflammatory programmed cell death resulting from inflammasome activation and is a new member of an ever-growing catalog of death processes whose biology is just now being unraveled [42]. As currently defined, canonical pyroptosis is a form of programmed cell death that is dependent on the activation of the enzyme caspase-1. In response to signals from pathogens or danger molecules, sensor proteins, including nucleotide-binding domain and leucine-rich repeat-containing (NLR) proteins [43], HIN200 protein family members and RIG-I-like receptors [44], can initiate inflammasome assembly by recruiting adaptor proteins. These inflammasomes then recruit and induce the autoproteolytic maturation and activation of caspase-1, which in turn cleaves and activates the pyroptotic substrate GSDMD and the inflammatory cytokines IL-1β and IL-18 [45]. In contrast to canonical pyroptosis, the noncanonical form of pyroptosis is mediated by caspases -4 and -5, which directly cleave GSDMD to induce pyroptosis but do not cleave the inflammatory cytokines IL-1β and IL-18 [46]. Since pyroptotic cell death is coupled with foreign invader detection and immune activation, we investigated the activation of pyroptosis in response to implant debris and determined whether it had significant functional relevance in wear particle-induced inflammatory osteolysis. We found that during inflammatory osteolysis, osteocytic pyroptosis was markedly increased in response to UHMWPE particle stimulation, as evidenced by the increased protein levels of active caspase-1, IL-1β, and GSDMD, as well as the increased secretion of mature IL-1β and the activity of LDH. Furthermore, our data revealed that inhibiting osteocytic pyroptosis with the caspase-1 inhibitor Ac-YVAD-CMK significantly impaired inflammatory cell infiltration, IL-1β secretion, osteoclast formation and bone resorption induced by UHMWPE particles. Since inflammatory cytokines, such as IL-1β, increase the expression of RANKL and/or have direct effects on osteoclastogenesis and the resorption of adjacent bony structures [47], it is reasonable to conclude that osteocytic pyroptosis facilitates osteoclast formation and bone resorption (Fig. 7), although the specific molecules derived from osteocytes that mediate osteoclast differentiation still need further investigation.

By understanding the pathogenesis of periprosthetic osteolysis, some effective preventative and nonsurgical interventions targeting osteoclasts have been introduced [32]. Since bone formation is linked to resorption through several coupling factors, antiresorptives targeting osteoclasts can decrease osteoblast activity [48]. Thus, identifying more suitable strategies for wear particle-induced osteolysis is urgently needed. We found that DFO could ameliorate UHMWPE particle-induced osteoclastic bone resorption by decreasing osteocytic pyroptosis. Osteocytic pyroptosis was significantly impaired by DFO and accompanied by decreased inflammatory responses and osteoclastic bone resorption. Since the deleterious effect of osteocytic pyroptosis on calvarial osteolysis has been observed, it is reasonable to conclude that DFO may ameliorate UHMWPE particle-induced osteolysis by decreasing the pyroptotic death of osteocytes (Fig. 7).

Our study suggested that UHMWPE particle induced pyroptosis in osteocytes and the release of inflammatory cytokines, including IL-1β, which facilitated osteoclast differentiation and led to osteoclastic bone resorption. However, it should be noted that a previous study demonstrated that osteocyte-like cells exposed to both polyethylene and metal wear particle types showed upregulated expression of the osteoclastogenesis markers RANKL and M-CSF [17]. In addition to direct resorptive effects, polyethylene wear particles upregulated the expression of genes associated with osteocytic osteolysis, including MMP-13, carbonic anhydrase 2, CTSK and TRAP, resulting in the loss of osteocyte perilacunar bone [49]. Consistently, we found increased RANKL and OPG expression in response to UHMWPE particles in the MLO-Y4 mouse osteocyte cell line (Fig. S1). Moreover, UHMWPE particle implantation significantly increased the number of empty osteocyte lacunae. However, whether the protective effect of DFO on calvarial osteolysis induced by UHMWPE particles by alleviating osteocytic osteolysis still needs further investigation.

However, we would like to point out some potential limitations of our study. First, although the mouse calvarial osteolysis model is widely used to examine the mechanisms of implant debris-induced osteolysis, mechanical loading may affect particle-induced osteolysis in patients with endoprosthetic surgery, which is not considered in mouse calvarial osteolysis [39]. Second, increased osteocyte death was detected in the UHMWPE-induced mouse calvarial osteolysis model. Using the mouse osteocyte cell line MLO-Y4, we found that UHMWPE particles significantly induced pyroptosis in osteocytes. It must be noted that other studies have shown that human osteocyte viability is unaffected by UHMWPE particles [17, 49], which suggests that the responses in the two species may be different. Third, the size of UHMWPE particles used to generate the mouse model was uniform, whereas UHMWPE particles from artificial joints are not identical, and particle shapes are believed to have a major influence on the survival of peri-implant cells [3]. Fourth, in addition to osteocytes, some other peri-implant cells, including osteoblasts, osteoclasts, and macrophages, are sensitive to implant debris [50]. Whether UHMWPE particles can induce pyroptosis in these cells still needs further investigation. Fifth, our results revealed caspase-1-dependent IL-β maturation and pyroptosis in osteocytes in response to UHMWPE particles. The activation of multiple inflammasomes, including NLRP3, NLRP1, NLRC4, and AIM2, has been reported to mediate the maturation and activation of caspase-1 [51]. Therefore, it remains to be determined which inflammasome is involved in the UHMWPE particle-induced pyroptosis in osteocytes.

## Conclusions

Taken together, our data demonstrated a potential pathogenetic role of osteocytic pyroptosis in UHMWPE particle-induced osteoclastic osteolysis. Furthermore, we uncovered a protective role of DFO against UHMWPE particle-induced calvarial osteoclastic osteolysis by decreasing osteocytic pyroptosis.

## Methods

### Preparation of UHMWPE particles

UHMWPE particles were provided by the manufacturer (Zimmer Inc., Warsaw, IN, USA). The characteristics of the particle’s morphology have been published previously [52]. The mean diameter of these particles is 2.6 μm (range from 0.7 to 21 μm). To avoid contamination with endotoxins, the particles were washed three times with 70% ethanol and sterilized for 72 h to remove endotoxin and heat sterilized, then dispersed in PBS at 2 × 10^8^ particles per ml. Endotoxin levels of the particle suspension were determined by a Limulus assay according to the manufacturer's instructions.

### UHMWPE-induced calvarial osteolysis model

The particles preparation and a wear particle-induced mouse calvarial osteolysis model was generated as previously described[39]. Briefly, 8-week-old C57BL/6 J mice were randomly divided into different groups. The mice were anesthetized, and the cranial periosteum was separated from the calvarium by sharp dissection. Then, 100 ul of particle suspension contained with or without Ac-YVAD-CMK (Sigma, 0.2 mg/ml), DFO (Sigma, 9 mg/ml) was uniformly spread over the periosteum at the middle suture of the calvarial. The mice were sacrificed 14 days after the operation, and the calvarial were excised for protein extraction, or fixed in 4% paraformaldehyde for micro-CT and histological analysis. No adverse events were found during the generation of mouse calvarial osteolysis model.

### Micro-CT imaging analysis

The fixed calvarials were analyzed using a micro-CT scanner (Skyscan 1172; Skyscan; Aartselaar, Belgium). All calvarials were scanned according to the same parameters (pixel size, 9 μm; X-ray voltage, 50 kV; electric current, 500 μA; rotation step, 0.7°). Serial tomographs were reconstructed from raw data using Conebeam reconstruction software (NRecon; Bruker). After reconstruction, a spherical volume of interest of 3 mm in diameter around the midline suture was selected for further qualitative and quantitative analysis. Bone mineral density (BMD), bone volume against tissue volume (BV/TV) were measured. All measurements were performed according to the guidelines of the American Society for Bone and Mineral Research.

### H&E and TRAP staining

After micro-CT scanning, the samples were decalcified in 10% EDTA for 3 weeks and then dehydrated, embedded in paraffin. Histological Sects. (5 μm thick) were prepared for H&E and TRAP staining. TRAP staining was performed using a TRAP staining kit (CS0740, Sigma). The specimens were then examined and photographed under a high-quality microscope (ZEISS, Jena, Germany)[53].

### Histopathological diagnosis

Paraffin-embedded calvarial sections were used by H&E staining for the histopathological diagnosis, and the clinical histology score was evaluated as none (0), mild (1), moderate (2) or severe (3) according to the inflammatory cell infiltration and bone resorption, the analysis was performed using conventional optical microscopy (ZEISS, Jena, Germany). Image acquisition and analysis were performed blinded.

### Immunohistochemical and Immunofluorescence staining

The Immunohistochemical and Immunofluorescence analyses were performed as previous report [53]. 5 μm thick paraffin sections were deparaffinized, rehydrated and followed by treated with 3% hydrogen peroxide to inhibit endogenous peroxidase (30 min, 24 C). Unspecific antibody binding was blocked by incubating the sections in tris buffered saline (TBS) supplemented with 2% bovine serum albumin (w/v) for 30 min. The samples were then incubated with a primary antibody against caspase-3 (#9664, CST), caspase-1 (A0964, ABclonal, Wuhan, China) at 4 ℃ overnight. After washing with PBS, the samples were incubated with horseradish peroxidase-conjugated secondary antibody for 30 min at room temperature. The washed sections were then incubated with diaminobenzidine (DAB; Solarbio, DA1010). TUNEL detection was performed with the TUNEL Assay Kits (C10619, Thermo). Images were captured under a microscope (ZEISS, Jena, Germany). Apoptotic cells (TUNEL staining) were counted in 5 image fields/mouse and correlated to the total number of nuclei. Image acquisition and analysis were performed blinded.

### Protein isolation for western blot

After dissected the calvarial tissues from the mice, the periosteal surface was stripped from the calvarial by tweezers. After washed 3 times in PBS, the calvarial tissues were milled by FastPrep-24™ 5G (MP Biochemicals) and resuspend in RIPA buffer containing protease inhibitor. After lysing in RIPA buffer for 20 min on ice, the lysates were collected and centrifuged at 14,000 × g for 15 min and the supernatants that contained the proteins were harvested. To isolated protein from cultured cells, cells were washed 3 times with PBS followed by lysed in RIPA buffer on ice for 20 min. The lysates were collected and centrifuged at 14,000 × g to remove cell debris. Protein concentrations were determined with the BCA Protein Assay Kit (Beyotime, Shanghai, China).

### Western blot analysis

Western blot analysis was performed as previously described [48]. Each sample containing 10 μg of total protein was separated by SDS-PAGE in a 10% gel and transferred onto PVDF membranes (EMD Millipore Corporation, US). After blockage with 5% nonfat dry milk in Tris-buffered saline with 1‰Tween (TBST), the membranes were incubated overnight at 4 °C with primary antibodies against caspase-1 (#24,232, CST), IL-1β (#27,989, CST), GSDMD (#39,754, CST), β-actin (#58169S, CST). After three washes with TBST, the membrane was incubated with horseradish peroxidase–conjugated secondary antibodies (Jackson). The antibody–antigen complexes were visualized with Immobilon reagents (Millipore).

### RNA extraction and quantitative real-time PCR (qRT-PCR)

RNA extraction and qRT-PCR were performed as previous report [39]. The mouse primer sequences for Trap, Nfatc1, c-Fos, Rankl, Opg and β-actin were described in Table 1.

**Table 1:** Primer sequences for real time-PCR

Gene		Primer sequence(5’- 3’)
Trap	FORWARD	CACTCCCACCCTGAGATTTGT
	REVERSE	CATCGTCTGCACGGTTCTG
C-Fos	FORWARD	CGGGTTTCAACGCCGACTA
	REVERSE	TTGGCACTAGAGACGGACAGA
NFATc1	FORWARD	GACCCGGAGTTCGACTTCG
	REVERSE	TGACACTAGGGGACACATAACTG
Rankl	FORWARD	CAGCATCGCTCTGTTCCTGTA
	REVERSE	CTGCGTTTTCATGGAGTCTCA
Opg	FORWARD	ACCCAGAAACTGGTCATCAGC
	REVERSE	CTGCAATACACACACTCATCACT
β-actin	FORWARD	GGCTGTATTCCCCTCCATCG
	REVERSE	CCAGTTGGTAACAATGCCATGT

### Organ culture and IL-1β detection

Organ culture and IL-1β detection was performed according to previous report [39]. The dissected calvarial tissue samples were weighted and cultured in serumless medium (10 ml/g weight) (Dulbecco’s Modified Eagles Media, Life Technologies, Gaithersburg, MD, USA) containing 1% Penicillin/Streptomycin for 72 h at 37 °C with 5% CO_2_. The release of IL-1β from dissected murine calvarial into the medium was measured with the enzyme linked immunoassay (ELISA) kit of mice IL-1β (Duoset R&D Systems, Abingdon, UK) according to the manufacturer’s instruction.

### BMMs isolation and osteoclastic differentiation

Primary BMMs were isolated from the long bones of 8-week-old C57BL/6 J mice. Cells were cultured in a 100 mm dish with complete α-MEM medium for 16 h. Non-adherent cells were harvested and cultured with fresh medium containing 50 ng/ml M-CSF. Three days later, the adherent cells were harvested as osteoclasts precursors. These cells were then seeded and further cultured with complete α-MEM medium or culture medium of MLO-Y4 cells containing M-CSF (30 ng/ml) and RANKL (50 ng/ml) for 3–5 days. Cell culture media were replaced every two days until mature osteoclasts had formed. Next, cells were used for RNA extraction, or fixed with 4% paraformaldehyde for TRAP staining.

### Culture and treatment of osteocyte cell line MLO-Y4

MLO-Y4 cells (Chinese Academy of Sciences) were cultured in α-MEM (GIBCO) medium containing 10% FBS (GIBCO), 50 U.ml^−1^ penicillin and 50 ug.ml^−1^ streptomycin (GIBCO) under standard culture conditions.

Our previous studies reported the culture of osteocytes on collagen gel coated plants [13, 54]. To detect osteocyte pyroptosis induced by UHMWPE particles, the osteocyte and particle co-culture system was designed as below. UHMWPE particles (0, 25, 50, 100 μg per ml) were mixed with 1 ml type I collagen (Gibco, #A10483-01) to produce the UHMWPE-collagen gel. Cell culture plants were then coated with the UHMWPE-collagen gel. Osteocytes were seeded on UHMWPE-collagen gel-coated plants and cultured for different time points. Cell proteins were harvested for western blot. Supernatant of cell culture were collected by centrifuged at 3000 rpm for 15 min followed by IL-1β concentration detection, LDH activity assay or for BMMs stimulation.

To detect the effect of DFO on osteocyte pyroptosis induced by UHMWPE particles. Osteocytes were seeded on UHMWPE-collagen gel-coated plants and treated with or without 50 μM DFO for 24 h. Cell proteins were harvested for western blot. Supernatant of cell culture were collected for IL-1β concentration detection and LDH activity assay.

To detect the effect of caspase-1 inactivation on osteocyte pyroptosis induced by UHMWPE particles. Osteocytes were seeded on UHMWPE-collagen gel-coated plants and treated with or without 30 μg/ml Ac-YVAD-CMK for 24 h. Cell proteins were harvested for western blot. Supernatant of cell culture were collected for IL-1β concentration detection and LDH activity assay.

To detect the effect of osteocyte pyroptosis on osteoclastic differentiation of BMMs. Osteocytes were seeded on UHMWPE-collagen gel-coated plants and treated with or without 30 μg/ml Ac-YVAD-CMK for 24 h. Supernatant of cell culture were collected by centrifuged at 3000 rpm for 15 min. BMMs cultured in 24-well plants was treated with 200 μl of supernatant, and their osteoclastic differentiation were detected by osteoclastic genes expression, TRAP staining and bone resorption assay.

### Bone resorption assay and F-actin ring formation assay

The bone resorption assay was conducted as previously described [39]. Briefly, BMM cells were plated onto bovine bone slices in 96-well plates at a density of 1 × 10^4^ cells/well. The BMM cells were cultured with complete α-MEM medium or culture medium of MLO-Y4 cells supplemented with M-CSF (30 ng/ml), RANKL (50 ng/ml). Cell culture media were replaced every 2 days until mature osteoclasts had formed. The osteoclasts were removed from the bone slices by mechanical agitation and sonication after 10 days. Resorption pits stained with toluidine blue were photographed under microscope (ZEISS, Jena, Germany). Three view fields were randomly selected for each bone slice for further analysis.

To perform F-actin ring formation assay, osteoclasts were fixed with 4% paraformaldehyde for 15 min, permeabilized for 5 min with 0.1% Triton X-100, and incubated with rhodamine conjugated phalloidin (Invitrogen Life Technologies, Grand Island, NY, USA) for 30 min at room temperature and then washed extensively with PBS three times. The F-actin ring distribution was visualized using a fluorescence microscope (ZEISS, Jena, Germany).

### LDH activity assay

The culture supernatants of calvarial tissue or MLO-Y4 cell were collected and the LDH activity was detected using the LDH assay kit (Nanjing Jiancheng Biology Engineering Institute, Nanjing, Jiangsu, China) as previously reported [55]. Briefly, 25 μL cell supernatant and 25 μL substrate were mixed together and incubated at 37 °C for 15 min. Then 25 μL 2,4-dinitrophenylhydrazine was added into the samples and incubated at 37 °C for 15 min. Finally, 250 μL 0.4 mol/L NaOH solution was added and incubated at room temperature for 5 min. The absorbance was measured at 450 nm on a spectrophotometric microplate reader.

### Statistical analysis

All data representative of three independent experiments are present as mean ± S.E.M. We used two-tailed *t*-tests to determine significances between two groups. We did analyses of multiple groups by one- or two-way ANOVA with Bonferroni post-test of GraphPad prism version 5. For all statistical tests, we considered *P*-value < 0.05 to be statistically significant.

## Supplementary information

**Additional file 1: Fig.S1**. Rankl and Opg expression induced by UHMWPE particles. (A&B) The relative mRNA expression of RANKL and OPG in calvarial bone induced by UHMWPE implantation (*n*=3). (C&D) The relative mRNA expression of RANKL and OPG in MLO-Y4 cells induced by 50 μg/ml UHMWPE particles for 24 hours. *P<0.05, ***P<0.001. P-values were analyzed by two-tailed t tests.

## Data Availability

The datasets used and/or analysed during the current study are available from the corresponding author on reasonable request.

## Abbreviations

UHMWPE: Ultrahigh-molecular-weight polyethylene
DFO: Desferrioxamine
TNF-α: Tumor necrosis factor alpha
IL: Interleukin
H: Hours
CT: Computed tomography
BMD: Bone mineral density
BV/TV: Bone volume/tissue volume
H&E: Hematoxylin and eosin
TRAP: Tartrate-resistant acid phosphatase
GSDMD: Gasdermin D
LDH: Lactic dehydrogenase
M-CSF: Macrophage colony-stimulating factor
RANKL: Receptor activator of nuclear factor kappa B ligand
PGE2: Prostaglandin E2
