# Supplementary material for: Desferrioxamine alleviates UHMWPE particle-induced osteoclastic osteolysis by inhibiting caspase-1-dependent pyroptosis in osteocytes
Source: J Biol Eng. 2022 Dec 8;16:34. doi: 10.1186/s13036-022-00314-8 (PMC9733322; doi:10.1186/s13036-022-00314-8)
Supplement: Supplementary file 1 — Additional file 1: Fig.S1. Rankl and Opg expression induced by UHMWPE particles. (A&B) The relative mRNA expression of RANKL and OPG in calvarial bone induced by UHMWPE implantation (n=3). (C&D) The relative mRNA expression of RANKL and OPG in MLO-Y4 cells induced by 50 μg/ml UHMWPE particles for 24 hours. *P<0.05, ***P<0.001. P-values were analyzed by two-tailed t tests. [file 13036_2022_314_MOESM1_ESM.docx]

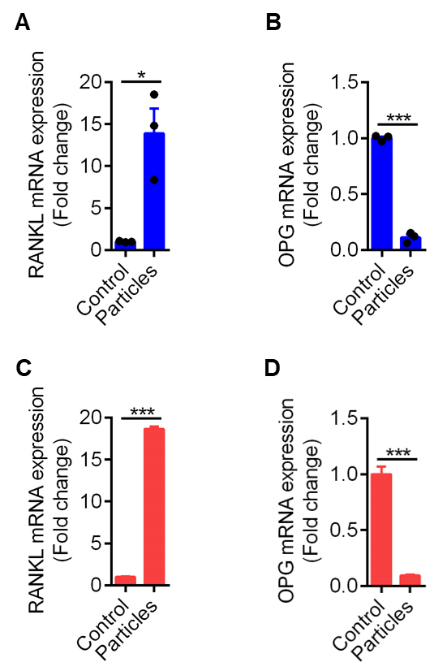


**Fig.S1. Rankl and Opg expression induced by UHMWPE particles.** (A&B) The relative mRNA expression of RANKL and OPG in calvarial bone induced by UHMWPE implantation (*n*=3). (C&D) The relative mRNA expression of RANKL and OPG in MLO-Y4 cells induced by 50 μg/ml UHMWPE particles for 24 hours. **P<0.05*, ****P<0.001*. *P*-values were analyzed by two-tailed *t* tests.
